# microRNAs in human brucellosis: A promising therapeutic approach and biomarker for diagnosis and treatment

**DOI:** 10.1002/iid3.519

**Published:** 2021-08-27

**Authors:** Sima Kazemi, Rasoul Mirzaei, Mohammad Sholeh, Sajad Karampoor, Fariba Keramat, Massoud Saidijam, Mohammad Yousef Alikhani

**Affiliations:** ^1^ Department of Microbiology, School of Medicine Hamadan University of Medical Sciences Hamadan Iran; ^2^ Department of Microbiology, School of Medicine Iran University of Medical Sciences Tehran Iran; ^3^ Gastrointestinal and Liver Diseases Research Center Iran University of Medical Sciences Tehran Iran; ^4^ Department of Virology, School of Medicine Iran University of Medical Sciences Tehran Iran; ^5^ Brucellosis Research Center Hamadan University of Medical Sciences Hamadan Iran; ^6^ Research Center for Molecular Medicine, School of Medicine Hamadan University of Medical Sciences Hamadan Iran

**Keywords:** biomarker, brucellosis, immunopathogenesis, microRNAs, therapeutic targets

## Abstract

**Introduction:**

Human brucellosis is a zoonotic bacterial disease with up to 500,000 new cases each year. The major evasion mechanisms from the host immune system by *Brucella* are restraint of complement pathway and Toll‐like receptors signaling pathways, interference with efficient antigen presentation to CD4‐positive T lymphocytes, selective subversion of autophagy pathways, inhibition of dendritic cell stimulation, inhibition of autophagolysosomal fusion, and macrophage apoptosis. Many molecular and cellular pathways contribute to brucellosis that microRNAs have a vital function in the immunopathogenesis of this disease. In this regard, these molecules apply for their roles by modulating various events like inflammatory reactions and immune defense. Recently, in the case of immunity to human brucellosis, it has been shown that microRNAs play an important role in immunity against these bacteria.

**Methods and Results:**

In this study, we tried to review the immune defense and immunopathogenesis of *Brucella* infection and highlight the current knowledge of the microRNAs in infected cells by *Brucella* pathogens. The recent findings suggest that the regulation of microRNAs expression is impaired during brucellosis infection, which may contribute to disease progression or inhibition by modulating immune responses against this pathogen.

**Conclusions:**

The interplay between miRNAs and *Brucella* pathogens and the underlying process required comprehensive examination to unravel the novel therapeutic or diagnostic approaches.

## Graphical abstract

Many molecular and cellular pathways contribute to brucellosis that microRNAs have a significant role in the immunopathogenesis of this infection. In this regard, these molecules apply for their roles by modulating various events like inflammatory reactions and immune defense. Recently, much insight into the significant role of microRNAs in immune protection in human brucellosis was found. In this study, we tried to review the immune defense and immunopathogenesis of *Brucella* infection and highlight the current knowledge of the microRNAs in infected cells by *Brucella* pathogens.

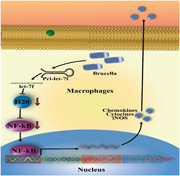

## 1 INTRODUCTION

*Brucella* genus, including *Brucella abortus*, *Brucella melitensis*, *Brucella canis*, and *Brucella suis*, is the etiologic agent of a zoonotic infectious disease called human brucellosis with up to 500,000 new cases annually.^1^, ^2^ Most cases were identified in the Middle East, Eastern Mediterranean, Mexico, the Arabian peninsula, South and Central America, India, and Central Asia.^2^, ^3^ Humans *Brucella* infection is defined by an undulant fever because of the fixation of infected macrophages in particular localization inside the body such as bones and spleen.^4^ Brucellosis causes some disorders like arthritis, meningitis, endocarditis, and osteomyelitis.^5^ *Brucella* bacterium is a facultative intracellular pathogen that resists killing by neutrophil cells. Also, this organism can replicate in macrophage cells “nonprofessional” phagocyte cells that cause a permanent interaction with the human cells.

Moreover, this reproduction feature of *Brucella* offers the capability to establish chronic and persistent infections.^6^ Hence, this organism is compatible with sophisticated host mechanisms to escape the host immune reactions. The central mechanisms in which *Brucella* evade from the immune system are the interference of complement system and Toll‐like receptors (TLRs) signaling pathways, interruption of efficient antigen presentation to T lymphocytes (CD4 positive), selective subversion of autophagy pathways, disruption in dendritic cell activation, inhibition of autophagolysosomal fusion and macrophage apoptosis.^7^, ^8^, ^9^, ^10^, ^11^, ^12^, ^13^, ^14^ In addition, it has been recently found that in brucellosis, the substantial roles of microRNAs (miRNAs) have a crucial role in immune evasion mechanisms.^15^, ^16^, ^17^, ^18^, ^19^, ^20^

Currently, it has been found that several miRNAs (including miR‐125b‐5p, miR‐21‐5p, miR‐ 23b, miR‐155, miR‐301a‐3p, mmu‐miR‐183‐5p, miR‐130a‐3p, miR‐146a, mmu‐miR‐199a‐3p, miR‐181a‐5P, and miR‐351‐5p and among others) have a role in the immunopathogenesis of Brucellosis.^16^, ^21^, ^22^, ^23^, ^24^, ^25^, ^26^, ^27^, ^28^, ^29^ For example, miR‐125 is a suppressor or promoter in various disorders and could induce the nuclear factor‐κB (NF‐κB) via targeting A20. In a study, Liu et al.^26^ noticed decreasing the levels of miR‐125b‐5p during *B. abortus* infection increased the A20 expression. In this regard, A20 so‐called tumor necrosis factor‐α (TNFα)‐induced protein 3 (TNFAIP3) regulates innate and acquired immunity, as well as a strong anti‐inflammatory, which interrupts some signaling pathways, stimulated via inflammatory cytokines as well as microbial pathogens.^30^, ^31^ The characterization of miRNA in *Brucella* infections has been an area of intense study; hence, in this review, the immune defense against *Brucella* and immunopathogenesis of brucellosis in humans are reviewed and discussed. Moreover, in the second section, the new information about miRNAs in brucellosis is summarized.

## 2 THE IMMUNE SYSTEM REACTIONS IN BRUCELLOSIS

The host immune reactions against *Brucella* have been commonly investigated in mouse models. Like their infection in humans, *Brucella* survives inside the mononuclear phagocyte in murine and can stay for a long‐term period in tissues in the absence of antibiotic therapy. In the primary step of infection, the host reaction resembles a T helper 1 (Th_1_) role, with interferon‐γ (IFN‐γ) formation by natural killers (NKs) and T lymphocytes.^32^, ^33^ In the host, CD4‐positive and CD8‐positive T lymphocytes can induce the control of *Brucella* infection, showing their activity as an origin for IFN‐γ formation.^34^ Transcriptional profiling of the primary immune reactions of the host found that the type four secretion system (T4SS) (encoded by *virB*) is critical for the pro‐inflammatory chemokines and IFN‐γ formation.^33^, ^35^ *virB* mutants cannot induce any inflammatory reaction.^33^, ^35^ It has been found that the *virB* T4SS triggers inflammation in brucellosis by translocating either conserved bacterial ligands as well as effectors of T4SS by cytosolic immune receptors of the host. The primary immune reaction toward *Brucella* is defined by the raised rate of pro‐inflammatory cytokines associated with Th1 roles like interleukin‐1β (IL‐1β), IL‐6, IL‐12, IFN‐γ as well as TNF‐α. Furthermore, it has been found that mutation in encoding genes of IFN‐γ, IL‐6, TNF‐α, and IL‐10 involves enhanced susceptibility against brucellosis.^36^, ^37^, ^38^, ^39^

Nevertheless, in chronic brucellosis, the primary Th1 role is suppressed and gains properties of Th2 actions like an enhancement in T lymphocytes forming IL‐13.^40^ Also, *B. abortus* triggers the anti‐inflammatory IL‐10^41^, ^42^, ^43^ and, interestingly, activated macrophages function by IFN‐γ formation toward *Brucella*‐like bactericidal capacity as well as the construction of pro‐inflammatory mediators were suppressed by IL‐10 during infection.^41^, ^44^ Experimental research found that IL‐10 formation by CD4‐positive CD25‐positive T lymphocytes was significant for macrophage function modulation during primary infection of *Brucella* because the murine lacking formation of IL‐10 by T lymphocytes as well as lacking the existence of the IL‐10 receptor in macrophage cells showed diminished bacterial survival in the liver, spleen, and enhanced formation of pro‐inflammatory cytokines as well as pathology in affected organs.^43^ Taken together, much work in human and murine immune systems in brucellosis will much be demonstrated to be worthy and equivalent in inducing the orchestration of the human reactions in *Brucella* infection.

## 3 microRNA BIOGENESIS AND ROLE

**Figure 1:**
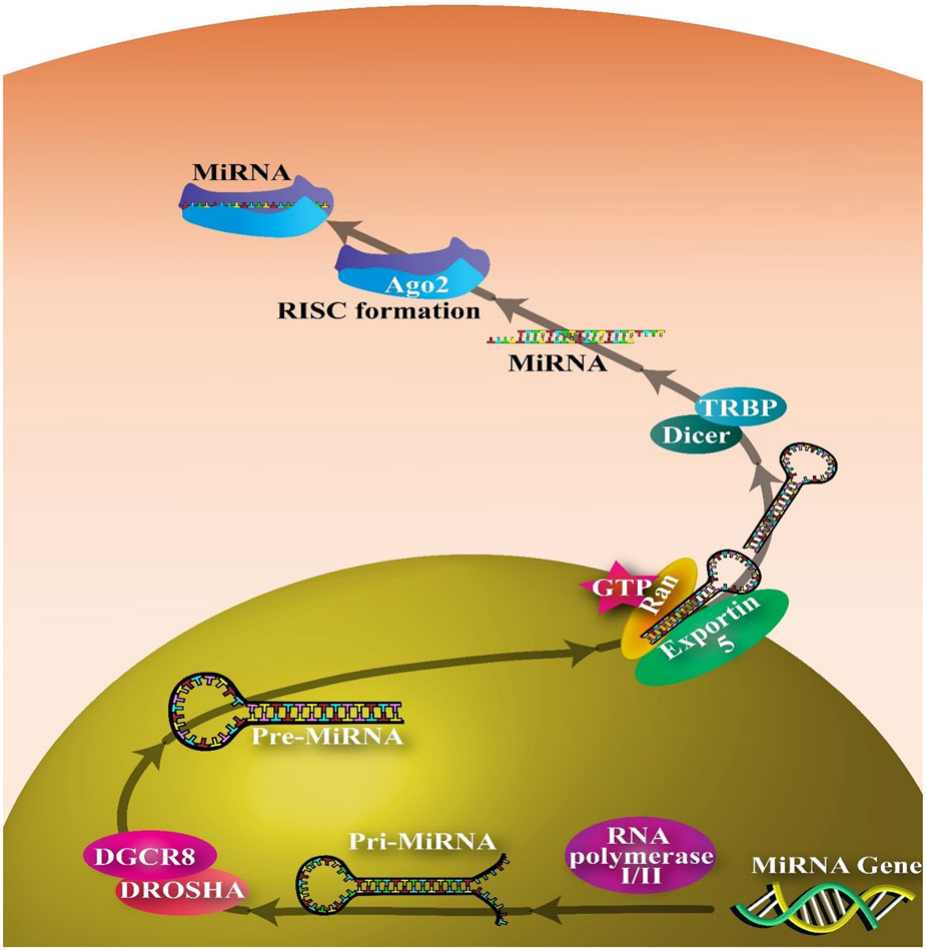
The pathway of microRNA biogenesis. The miRNA is transcribed by RNA POL 2 to a pri‐miRNA. The pri‐miRNA cleavage is done by RNase III endonucleases Drosha and RNA binding protein, DGCR8 (overall so‐called microprocessor complex). This molecule is translocated from the nucleus by the Exportin5 (EXP5) binding RAN‐GTP. Once guanosine triphosphate (GTP) is hydrolyzed can liberate the pre‐miRNA molecule into the cell cytoplasm, which another RNase 3 type enzyme (Dicer) attaches the 5ʹ end of the pre‐miRNA molecule and cleaves the double‐stranded RNA. Twenty‐two nucleotides double‐stranded miRNA is recognized by AGO2 protein, and one strand is preferentially loaded into a miRISC that targets conserved the 5ʹ end of the miRNA named the seed sequence where they guide RISC to silence target mRNAs via mRNA cleavage, deadenylation as well as translational repression, whereas the passenger strands are degraded. PASHA, the microprocessor complex Drosha–DGCR8; AGO2, argonaute 2; miRNA, microRNA; RISC, RNA‐induced silencing complex

The miRNA biogenesis is a regulated mechanism (Figure 1), and its characterization is described in detail in previous studies.^45^, ^46^, ^47^ The capacity of miRNA molecules to coordinate precise protein formation during differentiation, proliferation, inflammation, and apoptosis has found their significant activities in the host.^48^, ^49^, ^50^ Additionally, miRNA has been seen as new regulators of various pathways contributing to host immune responses, cancer, inflammatory, and autoimmune diseases.^20^, ^51^, ^52^, ^53^, ^54^, ^55^, ^56^, ^57^ It has been found that miRNA can have a seminal role in the expansion of host immune reactions. In some cases, it could play a negative feedback agent that influences and regulates immune responses.^47^, ^58^

## 4 microRNAs IN HUMAN BRUCELLOSIS

Currently, several miRNAs such as miR‐125b‐5p, miR‐369‐5p, miR‐586, miR‐520f‐3p, miR‐4307, miR‐505‐3p, miR‐4516, miR‐2861, miR‐3960, miR‐126‐5p, miR‐4753‐5p, miR‐181b, miR‐1981, miR‐7‐2‐3p, miR‐15a‐3p, miR‐103b, mmu‐miR‐199a‐3p, mmu‐miR‐183‐5p, miR‐130a‐3p, miR‐181a, miR‐301a‐3p, miR‐146a, and miR‐351‐5p have been found that have a role in the immunopathogenesis of brucellosis.^16^, ^21^, ^22^, ^23^, ^24^, ^25^, ^26^, ^27^, ^28^, ^29^ In the following, we overview these miRNAs in brucellosis.

### 4.1 microRNAs in acute and chronic brucellosis

Budak et al.^22^ evaluated the immunological agents related to CD8‐positive T lymphocytes and their activities in transmitting acute to chronic brucellosis. In their work, the regulatory activity of 2000 miRNAs in human CD8‐positive T lymphocytes was assessed, and it has shown 42 miRNAs in *Brucella* infection were involved, and two miRNAs were specifically formed in the chronic phase of brucellosis, and five miRNAs were formed in the acute phase of brucellosis.^22^ They showed that expression of miR‐369‐5p, miR‐ 4307, miR‐586, miR‐520f‐3p, and 505‐3p were reduced, and the formation of 37 miRNAs (such as miR‐4516, miR‐2861, and miR‐3960) was enhanced in both acute and chronic phases compared to the controls, respectively.^22^ Also, in this study, miR‐126‐5p and a miR‐4753‐5p significantly reduced CD8‐positive T lymphocytes in chronic brucellosis patients.^22^ Different miRNAs are contributed to mitogen‐activated protein kinase (MAPK) signaling, endocytosis, cytokine to cytokine receptor interplay, focal adhesion, and actin cytoskeleton regulation.^22^ Acute brucellosis is the destruction of bacterial cells by the host immune reaction. Unfortunately, failure to clear the infection leads to chronic brucellosis, which is defined as weight loss, sweating, mild fever, and so on. Chronic brucellosis has occurred in 10% to 30% of cases, notwithstanding primary determination and therapy. It is principally based on symptoms and clinical findings and the consequence of high immunoglobulin G (IgG) titers. Nevertheless, serological assays retain a special characteristic in determining brucellosis since IgG titers can persist for a long time after entirely signs are removed.^59^, ^60^, ^61^ In the work of Budak et al.,^21^ miRNA‐339‐5p, miRNA‐22‐5p, miRNA‐1914‐3p, miRNA‐575 as well as miRNA‐335‐5p were involved in *Brucella* infection and had comparable expression patterns in the severe and chronic groups than the control group, and 15 miRNAs were notably distinct between the severe and chronic groups. Among these, the formation of 14 miRNAs decreased, and the expression of miR‐125b‐5p in the chronic group increased than the acute group.^21^ The current study showed in the chronic model that the expression levels of miR‐1238‐3p were increased, while the levels of miR‐ 494, miR‐139‐3p, and miR‐6069 were declined.^21^ Also, miR‐139‐3p was mediated to the cell adhesions, chemokine signaling, bacterial attack of epithelial cells pathways, endocytosis, T lymphocyte receptor, arrangement of the actin cytoskeleton, cytokine–cytokine receptor communication, and leukocytes trans‐endothelial immigration, and MAPK signaling pathway, among others.^62^ Additionally, miRNA‐494 was linked to several involvements, including T lymphocyte receptor (TCR) signaling, TGF‐*β* signaling, organization of actin cytoskeleton, complement cascades, cytotoxicity associated to the natural killer, Fcγ‐R‐mediated phagocytosis, cell‐cycle, chemokine signaling pathway, apoptosis, phagosome, as well as MAPK signaling pathway in chronic brucellosis.^63^ miR‐6069 has a role in reorganizing the actin cytoskeleton, endocytosis, chemokine signaling, receptor interaction between cytokine to cytokine, MAPK signaling, TCR signaling, leukocyte transendothelial migration TLR signaling, FcγR‐mediated phagocytosis, the Janus kinase (JAK)–STAT signaling, and bacterial cell attack of epithelial cells signaling during chronic brucellosis^22^ and miR‐1238 have MAPK signaling, receptor interaction between cytokine to cytokine, organization of actin cytoskeleton, cell adhesion molecules (CAMs), phagosome, chemokine signaling, leukocyte transendothelial migration, TGF‐β signaling, Fc**∈ **RI signaling, endocytosis, protein processing in the endoplasmic reticulum, apoptosis, JAK–STAT signaling, cell cycle, Fcγ R‐mediated phagocytosis, tight junction, TLR signaling, and T‐cell receptor signaling

### 4.2 miRNAs in inflammatory signaling

**Figure 2:**
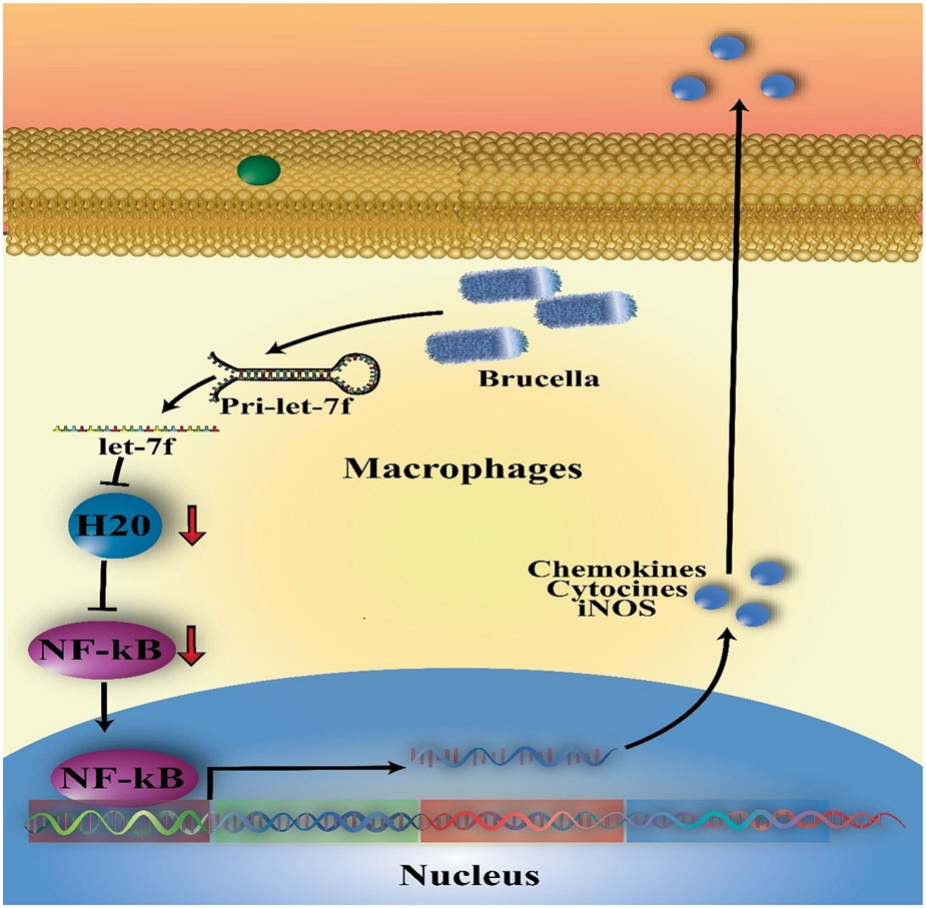
Let7‐7f expression by *Brucella* inhibits the formation of NF‐κB formation. It has demonstrated that *Brucella* pathogens have a particular composition that increased the formation of Let7‐7f in human cells. NF‐κB, nuclear factor‐κB

miR‐125, one of the most prominent miRNA families, is a suppressor or promoter in various disorders.^64^ This family includes hsa‐miR‐125b‐1, hsa‐miR‐125a, and hsa‐miR‐125‐2; recently, it has been demonstrated that miRNAs, such as miR‐125a and miR‐125b, could stimulate the NF‐κB via targeting A20.^64^ Besides, in macrophages infected with *Mycobacterium tuberculosis*, miR‐let‐7f inhibition has been shown to increase A20 target expression that causes reducing inflammatory signaling and facilitating bacterial survival (Figure 2).^65^ Liu et al.^26^ found that decreasing the expression of miR‐125b‐5p in *B. abortus* infection increased the A20 expression.^26^ A20 regulates innate and acquired immunity and is a strong anti‐inflammatory, interrupting some signaling pathways stimulated by inflammatory cytokines and microbial pathogens.^30^, ^31^ Luo et al.^27^ found that miR‐181a, miR‐301a‐3p, and miR‐130a‐3p regulated TNF‐α production by targeting the 3′‐untranslated region (3′‐UTR) region of TNF‐α, miR‐146a regulated TNF‐α expression by targeting tumor necrosis factor receptor (TNFR)‐associated factor 6 (TRAF6) and interleukin 1 receptor‐associated kinase 1 (IRAK1) (at the posttranscriptional level. In contrast, miR‐351‐5p accurately manages murine TNF‐α expression by targeting IRAK1 and TRAF6. Macrophages have an essential role in controlling *Brucella* infection via the generation of the IFN‐γ and TNF‐α.^66^, ^67^ It has been noted that TNF‐α is required for the full of anti‐*Brucella* activity of macrophages.^68^ TNF‐α depending on the quantity, time, and persistence of its formation by the host in reaction to *Brucella* disease and its significance in protecting *Brucella*, could be related to immunopathology. It has been found that the acquired immune reaction during Th1 lymphocytes may suppress or inhibit the growth of the organism that it induces macrophage to form additional radical oxygen and, also, other killing pathways by formation of TNF‐α and IFN‐γ.^69^ The signaling cascades mediated with TLR4 induction comprise targets of miR‐146: IRAK1 and TRAF6 and of miR‐155: TAB2. These cascades lead to NF‐kB nuclear translocation and stimulation of AP‐1 (that produces TNF‐α). In turn, TNF‐α involves the augmentation of the NF‐kB response via signaling of the TNF‐α receptor. Also, signaling of TLR‐4 may support other pathways in which stimulation of IRF transcription factors by Inhibitor‐κB kinase ε (IKKε), and TANK binding kinase 1 (TBK1) results in the formation of type I interferons. IKKε is prognosticated to be the target of miR‐155. Thus, a complicated net of miRNA‐155 and ‐146 feedback communications mediated to host response to brucellosis.^70^ miR‐155 is formed in activated B lymphocytes, T lymphocytes, macrophages, and DCs, and seems to be crucial for cell‐mediated immune reactions.^71^ Also, this miRNA is contributed to inflammation as a governor of macrophage activity, and it has found to have significant activity in the control of differentiation of T helper that causes in an optimal antibody action mediated to T lymphocytes, and this is partly because of the modulation of formation of cytokine.^72^ The miR‐155 formation is closely mediated to pro‐inflammatory transcription programs, responding within hours to appropriate stimuli.^70^ Additionally, miRNA‐146a is contributed to innate reactions by regulating the acute inflammatory activity after recognizing pathogens by TLRs on monocytes as well as macrophages. The miRNA‐146a then targets the formation of the following genes. IRAK1, TRAF6, IRF5, STAT1 and therefore has a negative regulator activity in TLR and INF‐γ signaling.^73^, ^74^, ^75^

### 4.3 miRNAs in TLR‐MyD88 signaling pathway

In a study by Corsetti et al.,^23^ it was shown that miR‐181a‐5p during *B. abortus* infection regulated miR‐21a‐5p, and TNF‐α which controls IL‐10 production. For miR‐21a‐5p, the GBP5 target gene was identified. The Omp25 is highly conserved among *Brucella* species. Some works have found that the use of the Omp25 or recombinant Omp25 DNA vaccine protects *B. melitensis* and *B. abortus*, making Omp25 a vaccine target. Also, it has been reported that Omp25 is contributed to the virulence of *Brucella ovis, B. melitensis*, and *B. abortus*.^76^, ^77^, ^78^ Whilst the mice therapy of susceptible *Brucella* with IL‐12 enhances primary and secondary immune response, the molecular mechanisms by which *Brucella* Omp25 hinders IL‐12 formation have not been well characterized.^67^ IL‐12 to form IFN‐γ, which induces the action of Th_1_ and stimulates macrophages. IL‐12 is one of the inherent inflammatory mediators that has a crucial activity in controlling intracellular bacterial infection, also is a central cytokine in the Th_1_ differentiation.^79^ Autophagy, as a prominent player in the intracellular innate immune system, controls some intracellular bacteria's fate via xenophobic capture, including nuclear machinery, ATG protein‐dependent, and elongation machinery.

### 4.4 miRNA in apoptotic and autophagic pathways

Zhang et al.^16^ in RAW264.7 cells were investigated miRNAs expression in response to *B. melitensis*. This study found that 344 miRNAs were co‐expressed in both mock‐ and RAW264.7 cells infected with *Brucella*. Also, the current study reported that the 57 miRNAs were increased following *Brucella* infection, among them miR‐1981, miR‐181b, miR‐142‐5p, miR‐151‐3p, miR‐92a, miR‐99a, miR‐93, and let‐7b were expressed distinctively.^16^ Gene Ontology (GO) enhancement analysis indicates that these miRNAs significant target genes contribute to apoptosis, autophagy, and immune reactions with different expressions.^16^ Specifically, a whole of 25 target genes participates in the regulation of autophagy and apoptosis, suggesting that specific miRNAs can perform a crucial regulative function in *Brucella–*host interaction.^16^ Zhang et al.^29^ identified 1372 miRNAs using Illumina sequencing via synthesis technology and 1893 new miRNAs identified in brucellosis. The miR‐15a‐3p, miR‐7‐2‐3p, and miR‐103b were upregulated in cases with brucellosis, that miR‐103b a significant increase in brucellosis patients compared to the healthy group. Further analysis showed that some of the mir‐103b targets are involved in apoptosis and autophagy.^29^ Cui et al.^24^ observed that the expression of three miRNAs, including miR‐ 23b, miR‐21‐5p, and miR‐155, were increased in outer core membrane protein (Omp25)‐expressing cells. Certain miR, such as miR‐23b and miR‐21‐5p, target the 3′‐UTR of *il12A* and *il12B* at the posttranscriptional level reduced IL‐12 p35 and p40 subunits expression. Another miR that can decline the expression of the IL‐12 p40 subunit via targeting 3′‐UTR of TAB2 is miR‐155.^69^ Jiao et al.^25^ in RAW264.7, macrophage infected with ΔOmp25 *B. melitensis* has identified networks of miRs (miR‐146a‐5p Dusp16p, mmu‐miR‐149‐3p‐ Ppp2r3c, and mmu‐miR‐149‐3p‐Ptpn5) were with mediated autophagic pathway. In a study, Rong et al.^28^ showed that two miRNAs of mmu‐miR‐183‐5p and mmu‐miR‐199a‐3p were upregulated in CD14 silenced RAW264.7 cells induced by *B. melitensis* infection. In this study, more than a thousand target genes were identified.^28^ These miRNAs and their target genes were associated with immune response and inflammatory reactions, stimulating innate immunity, apoptosis processes, antiapoptosis, cytokine formation, and cytokine‐mediated signaling.^28^ The Cbl‐b was introduced as a target gene for mmu‐miR‐199a‐3p in this study.^28^ These miRNAs are well associated with host genes that contributed to the innate immune response, inflammatory response, apoptotic, antiapoptotic processes, cytokine formation, as well as cytokine‐mediated signaling pathways.^28^ CD14 is a multifunctional receptor primarily expressed on monocyte and macrophage, with specificity for lipopolysaccharides (LPS), and performs an essential role in innate immunity.^80^ Bacterial cell wall parts, especially LPS, are detected by macrophages using the CD14 receptor.^81^, ^82^, ^83^ Activation of macrophages by CD14 causes the discharge of pro‐inflammatory cytokines like TNF‐α, IL‐1, and IL‐6 leading to the expression of acute‐phase reactants.^84^

## 5 miRNAs AS DIAGNOSTIC BIOMARKERS IN BACTERIAL INFECTION

It has been found that circulating miRNAs are contributed to the regulation of various reactions include the potential of predicting unhealthy conditions.^85^ Circulating miRNAs are easy to obtain without severe damage.^86^ In this regard, cell‐free miRNAs display crucial characteristics as biomarkers. First of all, miRNAs are stable in circulation as well as resistant to storage handling. For example, serum miRNAs are resistant to digestion by RNase and other situations like boiling, pH, and multiple freeze‐thaw processes.^87^, ^88^, ^89^ Besides, it has been found that various disorders and certain pathological situations mediate changes in miRNAs. Of note, some cell‐free miRNAs in body fluids can be packaged in microvesicles such as exosomes, which provide protection from degradation, result in transfer from one cell to another during various conditions.^90^, ^91^, ^92^, ^93^, ^94^ In summary, the accessibility and stability of cell‐free miRNAs make them valuable noninvasive biomarkers for infectious diseases.

Some works proposed that miRNAs act as a crucial regulator in microbial infection, demonstrating they are substantial‐excellent apply as new therapeutic factors. In this regard, some miRNAs were evaluated in trials; for example, in 2013, the leading drug based on miRNA entered stage 1 in patients with liver cancer was MRX34.^95^ The administration of miRNA‐based treatments faces come obstacles before they can be reached into clinical application for bacterial diseases. Even though no miRNA‐based drugs for bacterial disease have been assessed in clinical trials, miRNAs are an encouraging mechanism for tempering the immune response toward invading bacterial pathogens, such as miRNAs (such as miR‐146 and miR‐155) with powerful immune‐modulatory capabilities have a potential for further clinical studies in brucellosis. Tissue specificity, target delivery, and the endurance of drugs‐based miRNA are recent restricting agents for competent therapeutic impacts.^96^

*Brucella* infection causes serious human problems and causes significant economic losses in livestock.^7^, ^97^ Although there are some studies on *Brucella* infection in terms of miRNA, few studies have been evaluated the biomarker effects of serum miRNA expression in human brucellosis. In this regard, since serum and plasma are accessed with relative ease, circulating biomarkers are one of the most promising means of diagnosis. In a pioneer study by Zhang et al.,^29^ they investigated the differentially expressed miRNA profile in human brucellosis, and they performed a comprehensive analysis of miRNA expression with Illumina SBS technology and confirmed miRNA candidates by quantitative reverse transcription‐polymerase chain reaction. They found the three upregulated miRNAs (miR‐7‐2‐3p, miR‐15a‐3p, and miR‐103b) in patients, of which miR‐103b was found to be significantly and steadily increased in the brucellosis patients compared with the control group.^29^ Zhang et al.^29^ concluded that serum miR‐103b level markedly increases after *Brucella* infection and has the potential to serve as an additional diagnostic marker for human brucellosis combined with other existing laboratory tests like blood culture and serological tests that can improve the diagnostic accuracy for brucellosis. However, further investigations are required to survey the potential targets of miR‐103b and their relationship with the accurate occurrence and development of human brucellosis. Taken together, we recommend that all researchers interested in the detection and treatment of brucellosis move too many works in this field and use the miRNAs in this regard.

## 6 CONCLUSION

*Brucella* can establish chronic and persistent infection and has evolved with advanced strategies to control and evade the human immune system. The dominant strategies of *Brucella* for evasion of the immune response are interference with the complement system and TLR signaling pathways, impediment of efficient antigen presentation to T lymphocytes (CD4 positive), selective subversion of autophagy pathways, inhibition of dendritic cell stimulation, inhibition of autophagolysosomal fusion, and macrophage apoptosis. It has been found that miRNAs have a novel aspect for studying the process underlying the induction of host immune response to pathogens. As noted earlier, it has been appreciated that the miRNAs such as miR‐125b‐5p, mmu‐miR‐183‐5p, miR‐301a‐3p, miR‐21‐5p, miR‐351‐5p, miR‐23b, miR‐130a‐3p, miR‐146a, mmu‐miR‐199a‐3p, miR‐181a‐5P, and miR‐155 are well‐defined in the modulation of immunity and inflammation during brucellosis. The interplay between miRNAs and *Brucella* pathogens and the underlying process required comprehensive examination. Hence, the much characterization of the miRNAs in bost‐*Brucella* interplays could point to novel and preventive routes and the development of therapeutic strategies.

## CONFLICT OF INTERESTS

The authors declare that there are no conflict of interests.

## AUTHOR CONTRIBUTIONS

Sima Kazemi, Rasoul Mirzaei, Sajad Karampoor, and Mohammad Sholeh participated in the study design, wrote the draft, and collected the documentation materials. Mohammad Yousef Alikhani, Massoud Saidijam, and Fariba Keramat participated in the study to revise the draft. All authors read and approved the manuscript.
